# The Most Useful Drugs

**Published:** 1876-08

**Authors:** 


The Most Useful Drugs.—According to the *Medical Times and Gazette*, a party of ten medical men were dining together not long since, and one of them, during dessert, started the proposition that supposing all present were limited in their practice to a selection of six pharmacopœial remedies, which would be chosen as being most useful, compound drugs to be excepted. Each of the party wrote the names of the six drugs he should select, and handed to the doctor who started the proposition. On examining the lists it was found a majority of votes were given in favor of opium, quinine, and iron; between mercury and the iodide of potassium the votes were equally divided, as they were also between ammonia and chloroform.—*Canada Lancet.*

